# Are Physical Education Lessons Suitable for Sport Talent Identification? A Systematic Review of the Literature

**DOI:** 10.3390/ijerph17061965

**Published:** 2020-03-17

**Authors:** Alejandro Prieto-Ayuso, Juan Carlos Pastor-Vicedo, Sixto González-Víllora, Javier Fernández-Río

**Affiliations:** 1 Faculty of Education, University of Castilla-La Mancha, 16071 Cuenca, Spain; Alejandro.Prieto@uclm.es; 2 Albacete Balompié S.A.D., 02006 Albacete, Spain; 3 Faculty of Education, Universidad de Castilla-La Mancha, 02071 Albacete, Spain; JuanCarlos.Pastor@uclm.es; 4 Faculty of Education, University of Oviedo, 33005 Oviedo, Spain; javier.rio@uniovi.es

**Keywords:** sport, curriculum, gifted, school, talent development

## Abstract

Objectives: The goal of this study was to shed light on the existent knowledge, internationally published over the last decade (2009–2019), on how to deal with talented children in physical education (PE). Methods: A mixed systematic review (SR) was conducted following Preferred Reporting Items for Systematic Reviews and Meta-Analyses (PRISMA) guidelines and registered in the International Prospective Register of Systematic Reviews (PROSPERO), registration number: CRD42019117211. Study eligibility criteria: The articles included were selected using the following criteria: (a) studies published in peer-reviewed international journals; (b) studies published from 2009 to 2019 (both inclusive); (c) studies that included quantitative and/or qualitative methods and findings; (d) research conducted within school contexts; (e) articles that focused on both talent/gift and PE, and (f) studies published in English or Spanish. Results: A total of 11 articles were identified. Results showed a gradual change in both methods and instruments used for talent identification (TI) in PE, focused currently on children’s health and involvement in sports. Second, there is consensus on the lack of clarity in schools’ policies and guidelines on how to deal with talented children in PE. Conclusions: Finally, there are alternative programs to elite athlete models that better fit in PE to deal with talented children and to avoid child disengagement in PE and sports.

## 1. Background

The purpose of uncovering future sport stars has motivated many researchers to get into the field of sport talent detection. However, research into this topic has faced several difficulties [1] which currently remain. These difficulties lies in the ambiguity of the concept of sport talent [2] and the criteria commonly used for identification [3,4]. Research has been conducted in two directions: top-down and bottom-up. The first evaluates the characteristics of athletes who reached elite level, assessing their anthropometric characteristics [5], physical abilities [6], technical-tactical skills [7] or psychological traits [8]. The second aims to uncover the key points in the career of top athletes with the purpose of stablishing a vital itinerary that could help young individuals in their trail to excellence [9].

Regardless of the methodology considered (top-down or bottom-up), physical education (PE) has been suggested to be one of the possible contexts in talent identification and development [10], since it can provide children an early specialization which might be an indicator of later success [11,12]. Bailey and Collins [10], Bailey and Morley [13], Bloom [14,15] and Côté [16] suggested the important role of PE teachers when identifying talented pupils and implementing an appropriate program [17]. There are several examples around the world [18]: Talent eye and Talent Zurich in Switzerland; Youth Dance England and Excellence in Cities (EiC) in England; Winterball in Canada; Sporting Schools in Australia; and School Sports in India. Among all of them, EiC was one of the most important programs focused on talent in school. However, research highlighted some concerns related to the lack of clarity between PE and sport, and that PE teachers considered sporting performance solely as a criteria for detecting talented pupils [2,19,20]. Some voices have emphasized the difficult connection between PE and sport performance: “since many PE teachers play additional roles as coaches, it is sometimes difficult for them to draw the boundaries between those two jobs very clearly” (p. 104) [21]. Thus, Asland, Walseth and Engelsrud [22] and Croston and Hills [4] questioned who are the able and less able pupils in PE, as well as if those high-ability students are the gifted ones. This lack of differentiation between PE and sport could be caused by three views on sport initiation [23]: first, teaching methods that focus on results rather than methods; second, teaching game concepts in a vertical perspective or thematic approach; and third, the way in which the practice is conducted (health, competition or education).

There seems to be a wide range of both instruments and intervention programs for talented pupils in other curriculum subjects such as math or linguistics [24], but there are very few examples in PE. According to Bailey et al. [2], sports-based programs often ignore pupils who are potentially talented, but who, due to lack of opportunity or support, are currently underachieving, as well as those who excel in the other, non-performance aspects of PE, such as leadership, knowledge and understanding (p. 136). In 2006, Bailey and Morley [17] published their model of talent identification and development in PE, whose aim was to readdress the imbalance within the current debate from an almost total concern with out-of-school clubs and the reparation for adult elite sport in favour of a more equitable and inclusive approach, premised upon the unique importance of mainstream, curricular physical education within any talent development scheme (p. 211).

The model is based on abilities and outcomes (psychomotor, interpersonal, intrapersonal, cognitive and creative), talent development (discovery of potential performers, predicting performance, providing suitable learning, on-going process of identification), environmental characteristics, personal traits, access and opportunity. According to the authors, these categories can be used as a framework for supplementary studies, such as teachers’ evaluations of the relative significance of the different skills in PE, views of the relative influence of personal, environmental and genetic characteristics on talent, or systematic reviews (SR).

Regarding literature reviews on talent in PE, to our knowledge there are only two published articles. Gray and Plucker [25] tried to investigate the progress of research regarding athletic talent identification and development, including current issues, and provide suggestions for future research (p. 361). Fernández-Río and Méndez-Giménez [26] developed “a proposal on talent detection and nurturing in sport that considers physical education a key element” (p. 110). No literature reviews on the current status of talent in PE have been published since 2012, besides the recently published book of Baker et al. [27] dealing with the most important factors of talent identification and development, such as skill acquisition and motor learning, psychological factors and family influences. Nevertheless, the book is not focused on PE.

## 2. Objective

Given the paucity of studies in this area, the present article wants to shed light on the international literature published since 2009 and update the existent knowledge, reviewing the range of studies conducted on talent in PE internationally.

## 3. Method

### 3.1. Search Limits

A mixed systematic search [28] of five databases (Web of Science-All Databases, Scopus, SportDiscus, ERIC-Ebsco, and Academic Search Ultimate) was conducted (2009–2019). On the one hand, these databases were selected because they included PE articles published in journals indexed in the Journal Citation Report (JCR) or similar index (e.g., the Scimago Journal Rank-SJR). On the other hand, a mixed systematic search was conducted to obtain an extended examination of the phenomenon under study.

In addition, seven top-ranked journals in PE and giftedness, which are indexed in the databases subject to the SR conducted in this study (Physical Education and Sport Pedagogy, European Physical Education Review, Journal of Sports Sciences, European Journal of Sports Science, Gifted Child Quarterly, Journal for the Education of the Gifted, High Ability Studies), were reviewed. Table 1 shows the search strategy used in each database or journal.

The search was conducted following the Preferred Reporting Items for Systematic Reviews and Meta-Analyses (PRISMA) guidelines [29], including the PICO strategy: participants (e.g., primary, secondary, student, young), intervention (e.g., program, proposal), comparators (e.g., “Physical Education”, “sport context”), outcomes (e.g., detection, selection, development).

### 3.2. Selection Criteria

The criteria to include articles was as follows: (a) studies published in peer-reviewed international journals; (b) studies published from 2009 to 2019 (both inclusive); (c) studies that included quantitative and/or qualitative methods and findings; (d) research conducted within school contexts; (e) articles that focused on both talent/gift and PE, and (f) studies published in English or Spanish. Quantitative and qualitative articles were included in the SR for two reasons: (a) enlarge the sample, and (b) both types of research can play a role in research synthesis [30], maximising the strengths of both approaches and increasing the relevance of the SR [31]. Although calls have been made to further use and explore mixed-methods reviews in a systematic process, these methods are still not commonly used [32]. In short, a qualitative synthesis might be used to explore the findings of a prior quantitative synthesis or vice versa [33].

**Figure 1:**
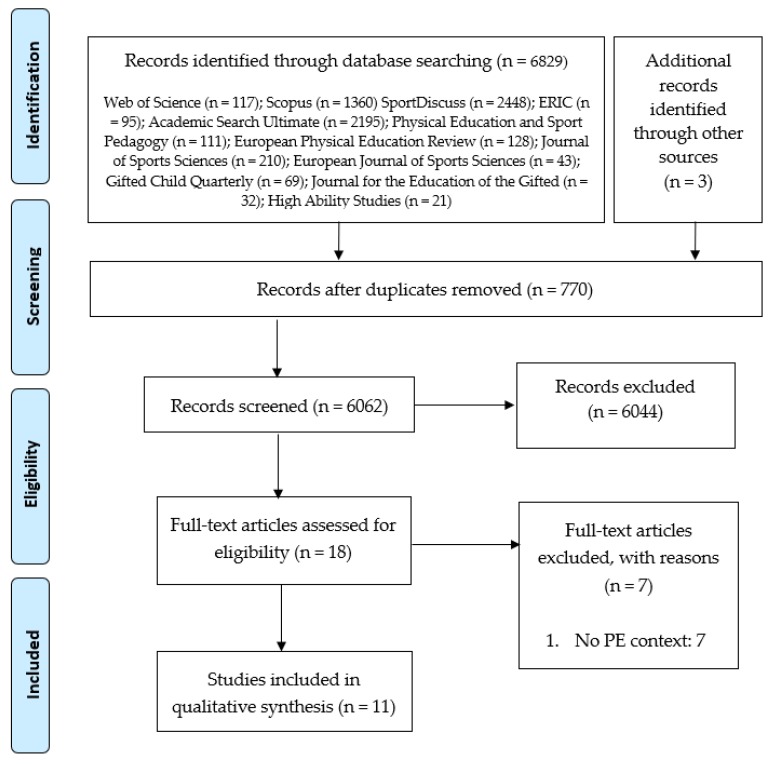
Flow diagram of the systematic search process.

Duplicated documents were disqualified at the first level of exclusion. At the second level of exclusion, documents were selected according to year of publication, title and abstract. Finally, at the third level of exclusion, the selected articles were fully read and some disregarded for the final analysis. Figure 1 shows the search process and the results found, following PRISMA [29].

After the identification and the screening, 6062 original articles were selected as potential studies. Then, 6044 were discarded at the second level of exclusion. After reading the full text of 18 articles, 11 articles formed the final sample.

### 3.3. Data Extraction and Reliability

The articles that did not fit the date of publication were discarded at the first level. The articles that met the selection criteria were retrieved for this review. In order to clarify the information, we classified the articles as follows [34]: authors; location; objectives; sample size; method; data sources; and results. Mendeley Reference Manager (Version 1803) was used to collect the documents from all the databases and filter the results.

### 3.4. Quality Assessent and Level of Evidence

First, the quality of the review process was assessed in the International Prospective Register of Systematic Reviews (PROSPERO) (https://www.crd.york.ac.uk/prospero/display_record.php?RecordID=117211) [35]. Second, the quality of the SR was also assessed using PRISMA guidelines [29] through an evidence-based set of items to report the quality of SR.

Third, the criteria for assessing the quality of the selected studies were based on the Checklist for Measuring Study Quality [36], the Strengthening the Reporting of Observational Studies statement [37], and the Consolidated Standards of Reporting Trials statement [38].

Fourth, previous studies [39,40,41] were used to obtain a quality score for each investigation based on the criteria shown in Table 2: journal, program, methodology, sample and instruments. Each item was scored from “0” to “2”. A total quality score from all the selected publications was calculated by adding up the number of positive items between “0” and “10”. Investigations were classified as: (a) low quality, a score lower than “3”; (b) moderate quality, a score between “4” and “6”; and (c) high quality, a score of “7” or higher. Four experts on physical education performed this evaluation independently. The selection criteria to be an expert included: (1) PhD in physical education and sport; (2) to be involved in at least one funded research project during the last five years (2015–2019), (3) to have published five articles indexed in the JCR or SJR in the last five years (2015–2019), (4) to have PE experience for a minimum of ten years. Cronbach’s alpha (0.95) indicated high reliability among the experts [39]. The reviews included in the final sample [10,25,26] were excluded from this evaluation because they could not be assessed using the selection criteria following a qualitative evaluation according to Chu and Zhang [40].

## 4. Results

A total of 11 studies were conducted internationally (see Supplementary Material, Table S1: Synthesis of studies about talent identification in Physical Education), from the first one [20] to the last one [42]. The relevant information was assessed following the structure used in previous SR [40]: ‘author(s)’ (and publication year); ‘focus’; ‘sample description’; ‘analysis/data sources’; and ‘outcomes’. A summary table with all the studies has been added as a supplementary material. Figure 1 shows the diagram flow followed during the search process.

### 4.1. Focus

During the last ten years, research on talent and PE has been conducted using descriptive studies through questionnaires or open-end interviews (4), reviews/proposals (3), intervention programs (3), and validation of instruments for talent identification (TI) in PE (1). Regarding intervention programmes, Faber et al. (2017) focused on a perceptual-motor skills program for talent detection in table tennis in PE lessons. Santos et al. (2017) identified the effects of the Skills4Genius sports-based training program. Finally, Lovell et al. (2017) examined the factors influencing selection in a school-based soccer program. Regarding reviews and proposals, Gray and Plucker [25] assessed athletic talent identification. Fernández-Río and Méndez-Giménez [26] developed a proposal on talent detection in sport that considers PE a key element. Lastly, Bailey and Collins [10] analysed the Standard Model of Talent Development. Regarding instruments for talent detection in PE, Hoeboer et al. [43] examined the validity of an Athletic Skills Track in a PE setting. Finally, concerning descriptive studies, Bailey et al. [20] investigated the ways in which schools identified and supported talented pupils in PE. Croston [44] drew a regional picture of practices and determined how PE teachers define talent in PE and sports. Lamb and Lane [45] evaluated pupils’ perceptions about being talented in PE. At last, Krombholz [42] evaluated the development of motor talents and non-talents at a preschool age.

### 4.2. Sample Description

Regarding each study’s context, England was the country with the largest number of studies (4) [20,44,45,46], followed by Germany (1) [42], Portugal (1) [47] and Netherlands (1) [43]. Four studies did not mention the context: one was a descriptive study [48] and the other three were reviews and proposals [10,25,26]. Concerning participants’ grade level, studies were conducted at all educational levels. However, the majority focused on primary education (3) [43,47,49], followed by secondary education (2) [45,48], and preschool (1) [42]. Only two studies were conducted with PE teachers [20,44]. Finally, the biggest sample (568 participants) was assessed by Krombholz [42] and the lowest (31 participants) by Lamb and Lane [45].

### 4.3. Data Resources and Analysis

Several types of data collection and analysis strategies were used: qualitative (3) [10,25,26], quantitative (5) [42,43,46,47,48], and mixed methods (qualitative and quantitative) (3) [20,44,45]. In the quantitative studies, motor performance tests (2) [42,47] and questionnaires were used (4) [20,44,45,46]. In the qualitative studies, no assessment instruments were used (3) [10,25,26].

### 4.4. Outcomes

The importance of PE for TI, based on its multifactorial nature, has been shown in all the studies reviewed [10,26,42,48]. Nevertheless, lack of policy in talent programs is a fact [20]. There seems to be consensus in the paucity of criterion in PE teachers on talent selection methods [25,44], focusing solely on sport performance [20]. PE teachers continue to use physical ability as a key indicator of talent [44]. However, researchers are currently conducting studies to validate other tools for TI in PE (AST) [43], assessing motor competence or perceptual-motor skills as a part of TI [46,47]. Finally, pupils seemed to positively perceive the support offered by mentoring programs [45] to fulfil their commitment to training and sport on the one hand, and the requirements of academic work on the other.

## 5. Discussion

The aim of this SR was to shed light on the scientific literature on talent in PE published since 2009 and update the existing knowledge. Results indicate that it is a potentially sensitive topic that deserves being studied the way it has been studied in other curriculum subjects such as math or linguistics [24]. The complexity of the current education landscape worldwide (e.g., curricula, accreditation requirements for teacher education programs, etc.) makes it impossible to visualize a unique picture. The debate around TI in PE has always been present. In the 1950s, PE was included in the school curriculum with sport as the main content [50], with PE being considered as the forge of the Olympic reserve [51]. PE and sport performance had a strong connection and the criteria for identifying the talented pupils in PE was exclusively based on physical elements and performance [50,52], despite limitations such as the lack of prediction and the low level of feedback provided [53]. However, since the 1980s the idea of PE as a suitable context for TI in sports has changed, giving health a more important role [54,55,56] and introducing a new concept, healthism: “the new health orientation in school physical education presents itself as an important site where these contributions are constantly being exposed” (p. 432). Later, this idea was supported by Kirk [55] who highlighted that the obesity problem had increased, and it is still present today in Quennerstedt [57], who focused in the connection between health and learning in PE.

Nowadays, the concept of TI in PE is wider than traditional perspectives linked exclusively to physical performance [58]. Collins et al. [15] presented a model of sport expertise in PE entitled *The Three Worlds Continuum*, where the goal is child engagement in sports regardless of the expertise level. The goal is to link the two cultures of elite sport and sport-for-all [59], and PE has a role to play.

Although current PE is not seen as the forge of Olympic reserve [51], there seems to be agreement in the literature reviewed about the role that PE should play as a stage in the pathway to excellence of talented pupils [10]. According to Kirk and Gorely [50], PE contexts can provide the fundamental motor skills that might be applied within a scale of competitive contexts. However, despite its importance, the paucity of published studies on TI focused on high ability and education [60] and on TI in PE [2].

From 2006 onwards, publications on talent in PE have increased. The model of talent in PE published by Bailey and Morley [17] was a breakthrough in the field. These authors studied the connections between talent and PE, highlighting that the implementation of programs in schools needs to be built on the foundation of quality PE. This placed PE at the same level of other curriculum subjects, where TI and enrichment programs are common [24]. Traditionally, when dealing with sports talent children are usually exposed to traditional programs in sport clubs [61]. Bailey and Morley [17] argued that this problem could be caused by the lack of criteria among PE teachers. These teachers tended to base their screening on physical condition tests only [58,62], with the risk that these kinds of criteria do not predict later success [53].

On the other end, new trends claim other methods of assessment for PE teachers, measuring not only physical condition, but also cognitive, emotional and tactical elements [63]. Hoeboer et al. [43] showed the validity of two skill tracks that evaluate motor competence in PE settings. Three years later, Bailey and Morley [20] conducted a national survey on policy and practices in England, concluding that a global new school policy is necessary because talented pupils were being identified by PE teachers based on current performance and not potential achievement. Thus, the performance in both school and club sport was the criteria used in order to identify talented pupils. Many teachers highlighted a shortage in competence for identifying talented pupils, and Croston [44] found similar results four years later. Based on these ideas, Gray and Plucker [25] highlighted that identification methods were often sporadic and lacked criterion. This, combined with the pupils’ view that there was a lot of tension between fulfilling their commitments to training and meeting the requirements of their academic work [45], revealed a poor connection between sport clubs and school policies.

Over the last few years, the context around TI in PE has changed. Faber et al. [46] conducted a program for evaluating the perceptuomotor skills as part of TI for table tennis. Lovell et al. [48] assessed a school-based soccer program to evaluate the factors influencing selection into playing levels and playing positions. Santos et al. [47] implemented the Skill4Genius sports-based training program that “could be easily integrated into PE curriculum” (p. 13). These three programs focused on the observation of a combination of physical, perceptual, technical and tactical skills. Other publications used national surveys to know the teachers’ [44] and pupils’ [45] perceptions on TI in PE. Krombholz [42] reviewed the evaluation of motor talent and non-talent in preschool age (the only one on children 3–6 years). Results showed that children with high motor performance did perform better in coordination, fitness and manual dexterity compared to average or low performing children. It could help preschool teachers identify which children present global motor talent, not with the goal of specialising these children in a specific sport, but to provide them with a wide range of sports to help them develop their expertise [64].

The reviews published by Gray and Plucker [25], Fernández-Río and Méndez-Giménez [26] and Bailey and Collins [10] helped deepen this topic. Fernández-Río and Méndez-Giménez [26] summarized the most important aspects to consider when designing a system for talented children in PE in nine pillars (e.g., physical, psychological), while Bailey and Collins [10] analysed the Standard Model of Talent Development, where PE is the baseline of the pyramid, describing its weakness and strengthens.

It is possible to identify the change in the approach to TI in PE over the years, from a traditional perspective based on physical tests to a more contextual methodology based on cognitive, emotional, ability aspects, as well as involvement in sport and health. An example of the latter is the Elite Athlete Programs (EAPs) which have been introduced over the last couple of decades in Australia and New Zealand [1], although little research exists on how they are being built within PE. In order to keep this line of work at schools, it is advised to follow Brown’s recommendations which supports the implementation of some models that can fit better in PE to deal with talented children: the Multifunctional Pathways Model [65], the Alternative Model [50], the Tactical Games Model [66,67] or the Sport Education Model [68]. They are an alternative to elite athlete models that focus solely on competency and that perceive ability as fixed and static. In addition, the use of these models could avoid children disengagement in PE and sport [69], which is one of its main objectives in primary and secondary education [15].

Finally, based on this literature review on talent in PE, a brief list of recommendations could be the following: (a) PE teachers should focus TI on the improvement of health and the involvement in sport; (b) the subject’s status in the curriculum continues to deteriorate, similarly to 20 years ago [70], which makes advances on talent in PE difficult. Therefore, policy makers must put all the new trends/findings into practice, including the national curriculum indications on how to deal with talented children in PE: procedures for identification and development; (c) pre-service PE teachers should receive more training on talent identification and development.

As limitations of this study, the sample size could be bigger. However, more studies that met the inclusion criteria were not found. Future studies could extend this period in order to make the sample bigger. Moreover, the period has been restricted to the last ten years. Finally, all the studies were based on male samples.

## 6. Conclusions

The goal was to provide an overview of the international literature on talent in PE. First, it is possible to conclude that there has been a gradual change, both in methods and instruments used for spotting TI in PE settings from a traditional (based on performance) to a contextual perspective (that considers health, involvement in sport, individuals’ maturation, competence, psychological and physiological factors). Second, there is consensus on the lack of clarity in school policies and guidelines about how to deal with talented children in PE. Third, schools and sports clubs must be coordinated on how to identify these types of children. Finally, there are alternative programs to elite models (that focus solely on competency), that fit better in PE to deal with talented children and that try to avoid children disengagement in PE and sport, a golden goal.

## Figures and Tables

**Table 1:** Search strategy of each database.

Key	Database/Journal	Search Strategy
D	Web of Science (all databases)	(gift * OR talent * OR “high ability” OR “above average”) AND (“Physical Education” OR “school sport”) AND (detection OR selection OR identification OR development) AND (program OR intervention OR test OR proposal OR model OR framework OR guideline) AND (primary OR secondary OR student OR child * OR adolescent * OR young OR youth)
D	Academic Search Ultimate	
J	Physical Education and Sport Pedagogy	
J	European Physical Education Review	
J	Journal of Sports Sciences	
J	European Journal of Sports Sciences	
J	Gifted Child Quarterly	
J	Journal for the Education of the Gifted	
J	High Ability Studies	
D	Scopus	(gift * OR talent *) AND (“Physical Education”) AND (detection OR identification OR development) AND (proposal OR framework OR guideline) AND (primary OR secondary OR student OR child * OR adolescent * OR young OR youth) AND (program * OR Intervention OR model)
D	SportDiscus	
D	Academic Search Ultimate	

D = database; J = Journal.

**Table 2:** Quality score checklist.

Research	JCR/SJR	Study Description	Methods	Sample	Instr.	Total Score	Quality Level
Bailey et al. (2009)	2	2	2	2	2	10	HQS
Croston (2013)	2	2	2	2	2	10	HQS
Lamb and Lane (2013)	2	2	2	1	2	9	HQS
Hoeboer et al. (2016)	2	2	2	2	2	10	HQS
Faber et al. (2017)	2	2	2	2	2	10	HQS
Lovell et al. (2017)	0	2	2	2	2	8	MQS
Santos et al. (2017)	2	2	2	1	2	9	HQS
Krombholz (2018)	1	2	2	2	2	9	HQS

Notes: JCR/SJR inclusion (was the study published in a journal indexed on the JCR or SJR?). “0”, not indexed; “1”, indexed on SJR; and “2”, indexed on JCR; Study description (did the research offer a detailed description of the study?). “0”, not included; “1”, brief and undetailed description; and “2”, detailed description; Methods (did the paper report in detail the methodological process used?). “0”, not reported; “1”, reported but imprecise (not completely); and “2”, exhaustive description reported; Sample (number of participants). “0”, fewer than 10 participants; “1”, from 10 to 50 participants; and “2”, more than 50 participants; Instruments (did the research offer a detailed description of the instruments?). ‘-’, not applicable; “0”, not included; “1”, brief and undetailed description; and “2”, detailed description; JCR, Journal Citation Report; SJR, Scimago Journal Rank; HQS, high quality study; and MQS, moderate quality study.

## Supplementary Materials

The following are available online at https://www.mdpi.com/1660-4601/17/6/1965/s1, Table S1: Synthesis of studies about talent identification in Physical Education.
