# Supplementary material for: Are Physical Education Lessons Suitable for Sport Talent Identification? A Systematic Review of the Literature
Source: Int J Environ Res Public Health. 2020 Mar 17;17(6):1965. doi: 10.3390/ijerph17061965 (PMC7143044; doi:10.3390/ijerph17061965)
Supplement: Supplementary file 1 [file ijerph-17-01965-s001.pdf]

Synthesis of studies about talent identification in Physical Education.

Author(s)	Focus	Sample	Instruments	Analysis/data sources	Outcomes
Bailey et al. (2009)	Investigate the ways in which schools identify and support talented pupils in PE.	C: England<br>P: 535 PE teachers<br>L: -<br>A: -	Questionnaire with open-ended responses.	QUAL: successful talent development strategies, impact of current provision, future talent development.<br>QUANT: school policies, talent identification procedures, impact of current provision, departmental expertise and experience.	The most common criteria for assessment were reported to be performance in school sport and club sport.<br>The effectiveness and equity of the strategies may be compromised by a lack of policy direction, an uneven distribution of staff expertise (in favour of games activities) and a lack of focused professional development.
Gray and Plucker (2010)	Investigate the progress of research regarding athletic talent identification.	C: -<br>P: -<br>L: -<br>A: -	No instruments	QUAL: athletic talent defined, elements in talent identification and development, issues in the identification of athletic talent (predictability, age, talent versus practice), recommendations for youth workers.	Athletic talent is rarely identified with much accuracy, especially early in a child's development.<br>Talent selection methods are often sporadic, lack criterion, and those selecting are uneducated regarding identification of athletic talent.
Fernández-Río and Méndez-Giménez (2012)	Develop a proposal on talent detection in sport that considers PE a key element.	C: -<br>P: -<br>L: -<br>A: -	No instruments	QUAL: financial and human resources, integrated approach to policy development, foundation and participation, talent identification and development system, athletic	Physical, physiological, psychological, cognitive, sociological, perceptual, technical and tactical elements have been identified as influential on talent programs. PE has an enormous

				and post-career support, training facilities, provision and development of coaches, national and international competition structures, scientific research and sports medicine support.	potential for talent detection and nurturing in sport.
Bailey and Collins (2013)	Analyse the Standard Model of Talent Development.	C: -<br>P: -<br>L: -<br>A: -	No instruments	QUAL: the standard model of talent development, difficulties with the standard model, an alternative view: stressing development and inclusion.	The literature supports talent development that is multifactorial, allows opportunity for playful sampling of a range of sports during the early stages, progressively introduces time and resources necessary for sustained and addresses the gap between a child's potential and the player.
Croston (2013)	Stablish a regional picture of respective practices as well as determining how PE teachers define talent in PE and sport.	C: England<br>P: 84 PE teachers<br>L: Secondary<br>A: -	Questionnaire with open-ended responses	QUAL: definitions of talent in PE and sport (talent in PE, talent in sport).<br>QUANT: talent identification in PE (G&T policy, identification of talent, staff training).	PE teachers continue to predominantly utilise physical ability as a key indicator of talent, as well as inconsistencies in PE teachers' definitions of talent in PE and sport.
Lamb and Lane (2013)	Evaluate pupils' perceptions about being G&T in PE.	C: England<br>P: 31 pupils (17 male, 14 female)<br>L: Secondary	Interviews<br>Focus groups<br>Questionnaire	QUAL: Pupils´ views about being on the G&T register, pupils´ view on the demands of their academic work, pupils´	Pupils expressed a tension between fulfilling their commitments to training and sport on the one hand

		A: 10-11		views on academic. support available to them, pupils´ views on what could support them as G&T pupils, pupils´ views on their life beyond the G&T register<br>QUANT: year group, gender and main sport.	and meeting the requirements of their academic work on the other.<br>Support offered through individual mentoring was received positively.<br>However, they felt they would benefit from more support for their academic needs, especially in managing their workloads.
Hoeboer et al. (2016)	Examine the validity of an AST in a PE setting.	C: Netherlands<br>P: 463 children (211 girls, 252 boys).<br>L: primary<br>A: between 6 and 12.	KTK, AST-1 and AST-2	QUANT: FMS (see Hoeboer et al., 2016)	An AST might be a feasible alternative for the current motor competence tests to assess children's FMS in a PE setting.
Faber et al. (2017)	Evaluate a perceptuomotor skills program<br>for talent detection in table tennis in PE lessons	C: England<br>P: 267 children (146 talent and 121 non-talent).<br>L: Primary<br>A: between 8 and 10.	Perceptuomotor skills assessment<br>Questionnaire	QUANT: sprint, agility, vertical jump, speed while dribbling, aiming at target, ball skills, throwing a ball, eye-hand coordination, sex, height, weight, sports participation, type of sport and training per week).	The table tennis players outperformed their primary school peers on all "ball control" items.<br>The perceptuomotor skills assessment is only intended as part of a talent detection program to help children to find a sport that fits their personal preferences and accommodates them at the specific level of their abilities.

Lovell et al. (2017)	Examine the factors influencing selection in a school-based soccer program.	C:<br>P: 216 adolescents<br>L: secondary<br>A: between 10 and 16.	Tests<br>Coach ratings	QUANT: anthropometry, maturation, physical capacity, technical ability, motor competence and subjective rating of each player.	Individual characteristics influence the selection process in recreational youth soccer, which have important implications for talent development pathways in schools, clubs and academies
Santos et al. (2017)	Identify the effects of the Skills4Genius sports-bases training program.	C: Portugal<br>P: 40 children<br>L: primary<br>A: 9 and 10	Torrance Test of Creative<br>Motor performance<br>In-game creative behaviour	QUANT: fluency, elaboration, originality, resistance to premature closure and abstractness of titles. Vertical jump, sprint test and agility T-test. Individual behaviour (technical performance) and Collective behaviour (tactical performance).	The training program nurtures the fundamental motor skills and prepare children to read the game, as well as explore unusual technical-tactical behaviours.<br>This type of enrichment program could be easily integrated into the PE curriculum.
Krombholz (2018)	Evaluate the development of motor talents and non-talents in preschool age.	C: Germany<br>P: 568 children (288 boys and 280 girls).<br>L: Preschool<br>A: 3	Physical growth<br>Body composition<br>Motor and cognitive performance<br>Cognitive abilities<br>Questionnaire	QUANT: height, weight, BMI, skinfold thickness, coordination, physical fitness, manual dexterity, intelligence, verbal ability and concentration. Activities in sports clubs, socioeconomic status and immigration status.	Existence of a general talent than specific talent or non-talent and the importance of family or environment in pre-schoolers.<br>Talented children experiencing success in movement games and different motor skills will engage more in those activities and consequently gain more competence

PE, physical education; QUAL, qualitative data collect and analysis methods; QUANT, quantitative data collect and analysis methods, G&T, gift and talent; C, country; P, participants; L, level; A, age(s); AST, athletic skills track; KTK, körperkoordinationstest für kinder test; FMS, fundamental motor skills; BMI, body mass index
